# Comparative study of the antioxidant capability of EDTA and Irganox

**DOI:** 10.1016/j.heliyon.2023.e16064

**Published:** 2023-05-13

**Authors:** Dalal K. Thbayh, Marcin Palusiak, Béla Viskolcz, Béla Fiser

**Affiliations:** a Institute of Chemistry, University of Miskolc, 3515 Miskolc-Egyetemváros, Hungary; b Polymer Research Center, University of Basrah, Basrah, Iraq; c Higher Education and Industrial Cooperation Centre, University of Miskolc, 3515 Miskolc-Egyetemváros, Hungary; d Department of Physical Chemistry, Faculty of Chemistry, University of Lodz, 90-236 Lodz, Poland; e Ferenc Rakoczi II Transcarpathian Hungarian College of Higher Education, 90200 Beregszász, Transcarpathia, Ukraine

**Keywords:** DFT, HAT, IP, PA, Ethylenediaminetetraacetic acid

## Abstract

Oxidative stress makes it difficult to preserve food and negatively affect the applicability of polymeric packaging. It is typically caused by an excess of free radicals, and it is dangerous to human health, resulting in the onset and development of diseases. The antioxidant ability and activity of ethylenediaminetetraacetic acid (EDTA) and Irganox (Irg) as synthetic antioxidant additives were studied. Three different antioxidant mechanisms were considered and compared by calculating bond dissociation enthalpy (BDE), ionization potential (IP), proton dissociation enthalpy (PDE), proton affinity (PA), and electron transfer enthalpy (ETE) values. Two density functional theory (DFT) methods were used, M05-2X and M06-2X with the 6–311++G(2d,2p) basis set in gas phase. Both additives can be used to protect pre-processed food products and polymeric packaging from oxidative stress related material deterioration. By comparing the two studied compounds, it was found that EDTA has a higher antioxidant potential than Irganox. To the best of our knowledge several studies have been carried out to understand the antioxidant potential of various natural and synthetic species, but EDTA and Irganox were not compared and investigated before. These additives can be used to protect pre-processed food products and polymeric packaging and prevent material deterioration caused by oxidative stress.

## 1 Introduction

**Figure 1:**
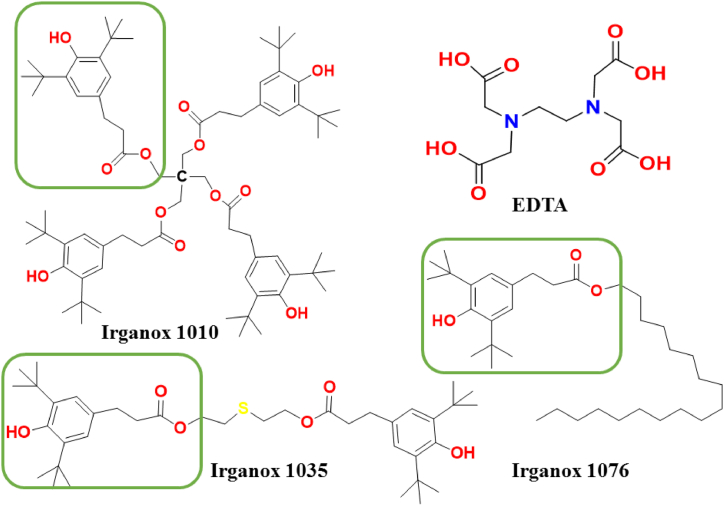
**2**D chemical structure of EDTA and various Irganox types which have the same base unit (highlighted in green frame) studied in the present work.

Free radicals can damage biologically important species in the body such as carbohydrates, proteins, lipids, DNA strains, cells, and tissues through the oxidative stress [1, 2]. Oxidative stress is dangerous to human health, resulting in the development of numerous diseases [3]. There are reactive oxygen (ROS), reactive nitrogen (RNS), and reactive sulfur species (RSS). These are responsible for many diseases such as cancer, cardiovascular diseases like atherosclerosis and stroke, neurological disorders, renal disorders, liver disorders, hypertension, rheumatoid arthritis and others [4, 5, 6]. The growing understanding of the dual nature of radicals has sparked a rise of interest in the compounds that might reduce excessive quantities of reactive oxygen, nitrogen, and sulfur species, hence limiting their damaging action. These substances, referred to as antioxidants, are a diverse set of molecules with the capacity to lessen oxidative stress [6, 7]. Antioxidants are compounds that may give free radicals an electron, which detoxifies them [8]. Antioxidants can be categorized in many ways based on their modes of action and physicochemical characteristics such as their solubility in different polar solvents. Based on their mode of action they can be classified as primary (chain breaking), secondary (preventive), and tertiary (fixer) antioxidants. Primary antioxidants are those which immediately interact with free radicals, leading to less reactive species or breaking the chain reaction. They are referred to be scavengers of free radicals. Secondary antioxidants provide protection without interacting directly with free radicals through mechanisms like metal chelation, initial antioxidant repair, decomposition of hydroperoxide into nonradical species, and absorption of UV radiation. Finally, tertiary antioxidants are those that have the capability to rehabilitate the oxidatively damaged biomolecules mostly by donating a hydrogen atom or an electron [7]. Depending on their source, antioxidant additives can be further categorized as natural and synthetic compounds. Natural antioxidants can be found in a range of natural products including fruits, leaves, and flowers [9, 10]. For example, curcumin is a natural antioxidant and commonly used as a yellow spice and it is derived from the dried rhizomes of turmeric (*curcuma longa*) [11] and it has been studied as an antioxidant additive before [12]. Ascorbic acid (vitamin C) is widely recognized as a potent antioxidant and free-radical scavenger [13]. There are many types of fresh fruits and vegetables rich in vitamin C like citrus fruits, kiwifruit, strawberries, papaya, blackcurrant, tomato, broccoli, and carrot [14] and other natural antioxidant additives. On the other hand, numerous synthetic antioxidants are frequently used as additives including butylated hydroxytoluene (BHT) [15, 16], *tert*-butylhydroquinone (TBHQ), butylated hydroxyanisole (BHA) [12], santowhite (SW) [14], propyl gallate (PG), ethylenediaminetetraacetic acid (EDTA) (Figure 1), Irganox 1010, Irganox 1035, and Irganox 1076 (Figure 1).

EDTA is an efficient metal chelating compound. It is also considered an efficient antioxidant and it can improve the oxidative stability, and some studies indicate that EDTA has the ability to protect against oxidation during storage [17]. The effect of EDTA as an antioxidant additive on the morphological, mechanical and water transport properties of polyurethane (PU) have been studied [18]. The addition of EDTA to PUs led to a good incorporation of the molecule into the polymer matrix and enhanced the final properties of the polymeric product [18].

Irganox is a family of compounds which are commercial hindered phenolic antioxidant additives (Figure 1). There are many types such as 1330, 1010, 1035, 1076, MD1024, 3114, E201, PS 800, 1081, and PS 802 [19,20]. Preservatives and packaging are both applied in food preservation. Antioxidant compounds including EDTA and Irganox types are important in both areas because they are applied as additives for various preprocessed food products and for polymeric packaging materials [21, 22, 23, 24, 25].

Although several studies have been carried out to understand the antioxidant potential of various natural and synthetic species, to the best of our knowledge, EDTA and Irganox were not compared and investigated before. In the present work, the antioxidant potential of EDTA and an Irganox model (Irg) (Figure 1) will be studied, and their applicability as synthetic antioxidant additives will be assessed by using computational tools.

## 2 Computational methods

To calculate the antioxidant potential of any molecules, three antioxidant mechanisms including hydrogen atom transfer (HAT), single electron transfer followed by proton transfer (SET-PT), and sequential proton loss electron transfer (SPLET) can be employed as these are described by the following equations (1-3) [[26], [27], [28], [29]].

(1) HAT: R^•^ + AH → RH + A^•^

(2) SET-PT: R^•^ + AH → R‾ + AH^+•^→ RH + A^•^

(3) SPLET: AH→ A‾ + H^+^ +R^•^ → A^•^ + R‾ + H^+^→ RH + A^•^

R^•^, AH, and A^•^ are radical, antioxidant, and antioxidant radical, respectively. AH^+•^ is radical cation created when an electron transferred from the antioxidant, A‾ is an anion which is formed after a proton loss has been occurred. By using the above equations, parameters such as bond dissociation enthalpy (BDE), ionization potential (IP), proton dissociation enthalpy (PDE), proton affinity (PA), and electron transfer enthalpy (ETE) can be determined and these can be used in the assessment of antioxidant potential of molecules [12, 14, 30, 31, 32]. From these parameters, we can evaluate the antioxidant activity of the molecules. To do all these calculation, we have been used Gaussian 09 software package for the studied molecules [33]. Two synthetic antioxidant additives including EDTA, model Irg, and the corresponding radicals, radical cations, and anions were computed via using two density functionals, M05-2X [34] and M06-2X [35] in combination with the 6–311++G(2d,2p) basis set in gas phase. The applied methodologies were previously validated in similar studies including thermochemistry, kinetics, and non-covalent interactions, especially those involving free radical reactions. Furthermore, because it has previously been utilized successfully in the study of similar systems and processes, the M06-2X/6–311++G(2d,2p) level of theory was chosen to be employed throughout the discussion in this article [12, 14, 26, 36, 37, 38, 39, 40, 41]. The structures' potential hydrogen donor sites (C–H and O–H) were all taken into account and compared.

In order to get insight into the bonding between individual atoms within molecules under investigation, we use the analysis of electron density according to Quantum Theory of Atoms in Molecules (QTAIM) [42, 43]. For that purpose, for the investigated systems, we estimated a mathematical representation of the electron density of selected molecular equilibrium geometries, using a wave function format in Gaussian (Output = WFX) at M06-2X/6–311++G(2d,2p). It is worth mentioning here that recent benchmark studies indicate the M06-2X functional as one which performs very well for QTAIM analysis [44]. Earlier studies of that type indicates 6–311++G(2d,2p) basis set as the one well performing in QTAIM analysis [45]. Such obtained data in WFX format was then processed according to QTAIM methodology, using the AIMAll software [46]. In the discussion section we focus on X–H bonding where X = C, O. We investigate the electron density, ρ, and its Laplacian, ∇^2^ρ, measured in bond critical points (BCPs, a characteristic point in molecule space being a local extreme of electron density function). Additionally, we analyse the delocalisation index (DI) estimated for X–H bonds and being the number of electrons shared by given two atomic centres (in other words, the number of electron pairs formed by those atomic centres). Delocalisation index can be interpreted as quantum-theory derived bond order, with that difference in respect to the classic bond order that it can be estimated for any possible atomic pair. In further discussion we analyse and comment only those DIs which were estimated for formally bonded atomic pairs of X–H type (X = C, O); thus, the bond-order-interpretation is fully allowed in this case.

## 3 Results and discussion

### 3.1 Geometrical considerations

**Figure 2:**
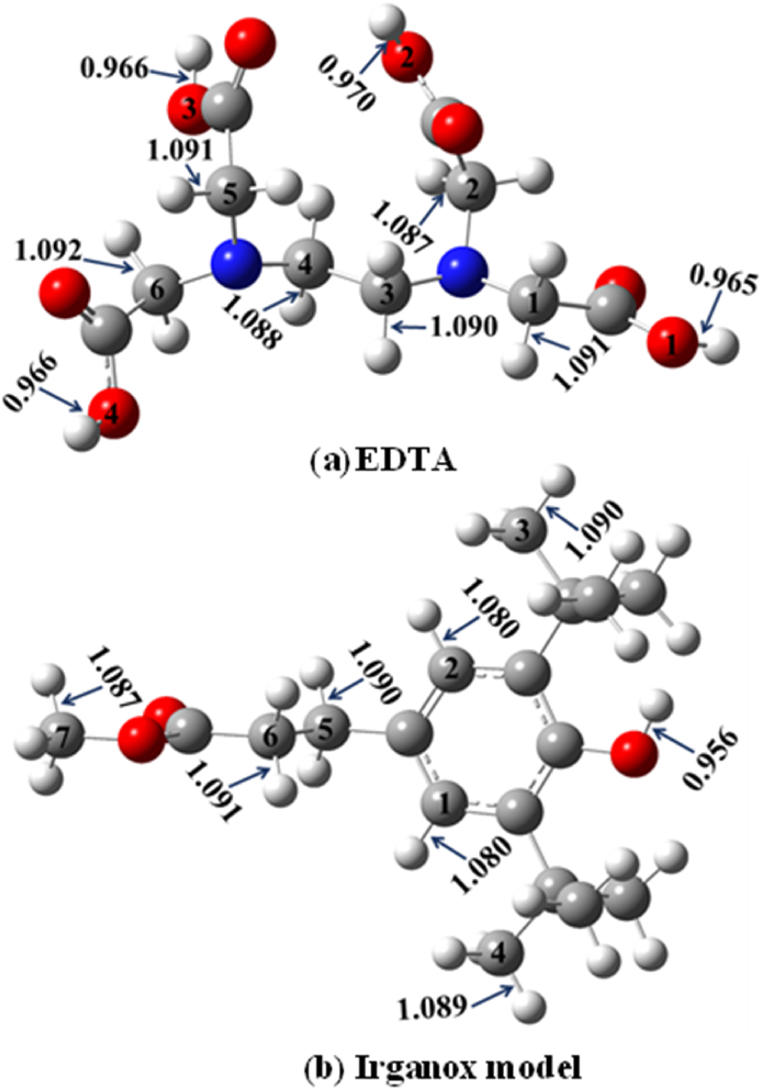
Optimized geometries of the studied synthetic antioxidant additives (a) ethylenediaminetetraacetic acid (EDTA) and (b) Irganox model. Geometry optimizations have been carried out at the M06-2X/6–311++G(2d,2p) level of theory in gas phase, and the corresponding bond lengths (in Å) are also shown.

EDTA has four carboxyl groups which have very similar O–H bonds, in terms of their length which are between 0.965 to 0.970 Å where the shortest one is O1–H and the longest one is O2–H while O3–H and O4–H are both equal to 0.966 Å (Figure 2). The carbon atoms which carry hydrogens in the structure are all directly linked to nitrogen atoms. The lengths of the bonds cover a narrow range from 1.087 to 1.092 Å where the longest is C6–H whilst the shortest is C2–H (Figure 2).

As for the Irganox model, the structure contains only one hydroxyl group with a bond length equal to 0.956 Å while the C–H bonds cover a range from 1.080 to 1.091 Å at the M06-2X/6–311++G(2d,2p) level of theory (Figure 2). The shortest C–H bonds are C1–H and C2–H with 1.080 Å and these are located on the benzene ring whereas the longest one belongs to C6–H.

### 3.2 Antioxidant mechanisms

The antioxidant properties of the two synthetic antioxidant additives including EDTA and the Irganox model have been studied by using two global hybrid functionals M05-2X and M06-2X in combination with the 6–311++G(2d,2p) basis set in gas phase. The antioxidant potential has been measured by comparing the two species in HAT, SET-PT and SPLET mechanisms.

#### 3.2.1 Hydrogen atom transfer (HAT) mechanism

The HAT mechanism involves a hydrogen atom transfer from hydrogen donor sites, such as methyl group, hydroxyl group and others, of the antioxidant compound to the free radical. From a thermodynamic point of view, the antioxidant potential can be measured by the bond dissociation enthalpy of the corresponding X–H bond, where the lower the BDE the greater the antioxidant capacity of the compound [6, 12, 14, 16, 29]. The BDE values for all unique X–H bonds of EDTA have been calculated and compared (Tables S1 and S2).

BDE values of the C–H bonds of EDTA computed at the M06-2X/6–311++G(2d,2p) level of theory, which cover a range between 316.7 to 367.2 kJ/mol. The weakest bond is C5–H while the strongest one is C3–H. In case of the M05-2X (Table S1), the weakest C–H bond is C6–H with a BDE equal to 313.1 kJ/mol, whilst the strongest is C3–H.

**Figure 3:**
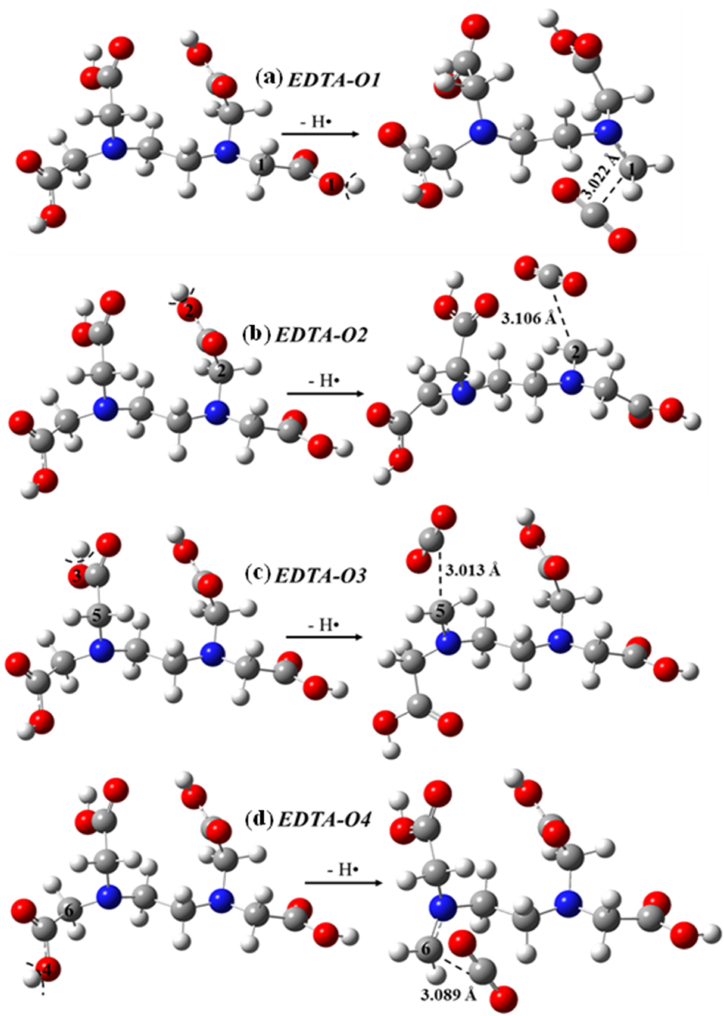
Optimized structures of ethylenediaminetetraacetic acid (EDTA) and the corresponding radical species after H-atom transfer in the hydrogen atom transfer (HAT) mechanism in case of the (a) O1–H (EDTA-O1), (b) O2–H (EDTA-O2), (c) O3–H (EDTA-O3), and (d) O4–H (EDTA-O4) sites. The species have been computed at the M06-2X/6–311++G(2d,2p) level of theory in the gas phase and specific geometrical parameters are also shown (in Å).

As for BDE values of O–H bonds, special situations arise in case of the carboxyl groups of EDTA. When the hydrogens were removed from the carboxyl groups large structural changes occurred (Figure 3 and Figure S3) as it induced a homolytic bond dissociation. The hydrogen atom abstraction from the carboxyl groups of EDTA initiated a successive C–C bond break, resulting in decarboxylation, a similar situation experienced before in the literature (Fiser et al., 2011). However, BDE values can be computed for O–H bonds, and these can be compared with the corresponding values of C–H bonds, but any conclusion must take into account the effect of the mentioned structural changes. The BDE of O–H bonds cover a range between 338.5 to 354.0 kJ/mol where the weakest bond is O4–H whilst the strongest is O1–H (Table S2) determined at the M06-2X/6–311++G(2d,2p) level of theory. Thus, O4–H has the highest antioxidant potential within the O–H bonds. The results with the other density functional are similar with only small deviations. But this is just an artefact of the position of the released carbon dioxide, as the four O–H groups should have identical bond dissociation values. As for the BDE values of the C–H bonds of EDTA computed at the M06-2X/6–311++G(2d,2p) level of theory, these cover a range between 316.7 to 367.2 kJ/mol. The weakest bond is C5–H while the strongest one is C3–H. In case of the M05-2X (Table S1), the weakest C–H bond is C6–H with a BDE equal to 313.1 kJ/mol, whilst the strongest is C3–H.

Therefore, by comparing the X–H bonds within EDTA, the order of antioxidant potential is the following: C3–H < C4–H < O1–H < O2–H < O3–H < O4–H < C1–H < C2–H < C6–H < C5–H. Thus, C–H bonds have higher antioxidant potential than O–H bonds within EDTA. At the same time, C3–H and C4–H have lower antioxidant potential and these bonds are located between two nitrogen atoms.

**Figure 4:**
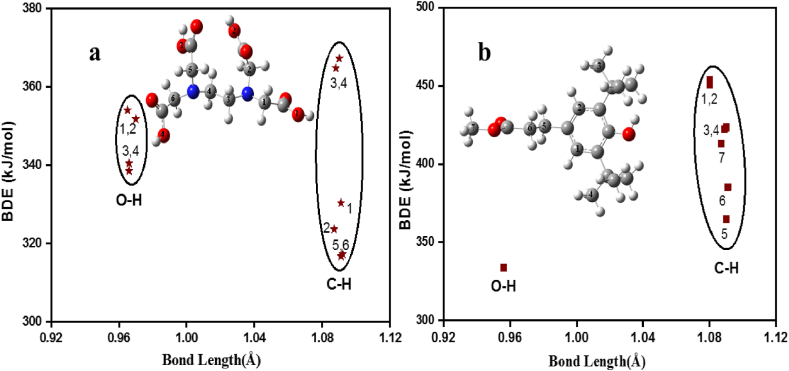
Bond dissociation enthalpy (BDE) *vs* bond length plot for two studied synthetic antioxidant additives (a) ethylenediaminetetraaceticacid (EDTA) and (b) Irganox model. The calculations have been carried out at the M06-2X/6–311++G(2d,2p) level of theory in gas phase. It has to be noted that the hydrogen atom abstraction from the carboxyl groups (O1–H, O2–H, O3–H, and O4–H) of EDTA initiated a successive C–C bond break, resulting in decarboxylation.

In the case of the Irganox model, the BDE values of the studied X–H bonds have also been calculated and compared (Table S3, Table S4 and Figure 4(a, b)). There is only one hydroxyl group in this structure and the corresponding BDE is 331.7 and 333.8 kJ/mol calculated with M05-2X and M06-2X, respectively. BDEs of the C–H bonds cover a range from 364.8 to 454.0 kJ/mol (Table S4), and the weakest C–H bond is C5–H whilst the strongest one is C1–H, a benzylic hydrogen. The O–H and C5–H are the most potent antioxidant sites within the model compound.

By comparing the results of the two synthetic antioxidant additives, it can be concluded that EDTA has a higher antioxidant potential than Irganox. To assess the applicability of these compounds as antioxidant additives, experimental BDE values of regularly used polymers (from 393.7 to 406.2 kJ/mol) [12, 14, 47] were compared to the lowest bond dissociation enthalpies (316.7 and 333.8 kJ/mol) of the studied species. It was found that both species can be used to protect polymers and prevent material deterioration caused by oxidative stress [12, 14, 47]. It must be noted somehow that some of the polymers compared applied in food packaging.

#### 3.2.2 Single electron transfer proton transfer (SET-PT)

The second studied mechanism was SET-PT which includes two steps. First an electron is donated from the antioxidant to the radical which is followed by proton transfer [12, 14]. In this case, the antioxidant potential is determined by the ionization potential (IP) and proton dissociation enthalpy (PDE) values. The lower IP and PDE values are associated with higher antioxidant capability of the additives [6, 12, 14, 15, 28]. The IP for EDTA is slightly above 700 kJ/mol (Table 1 and Table S5). PDEs indicates that C–H bonds in some positions in the structure are more prone to deprotonation in the second step of the SET-PT mechanism than O–H bonds except C3–H and C4–H. Thus, C–H bonds in EDTA have higher antioxidant potential than O–H bonds according to their IP + PDE values which is in good agreement with the results of the HAT mechanism.

**Table 1:** The ionization potential (IP), proton dissociation enthalpy (PDE), proton affinities (PAs) and electron transfer enthalpies (ETE) values in kJ/mol for ethylenediaminetetraacetic acid (EDTA) and Irganox model calculated at the M06-2X/6–311++G(2d,2p) level of theory in gas phase.

Compound	IP	PDE	IP + PDE	PA	ETE	PA + ETE
**EDTA**	700.1					
O1–H		956.0	1656.1	1410.0	255.1	1665.1
O2–H		947.5	1647.5	1325.2∗	380.1∗	1705.3∗
O3–H		950.9	1651.0	1300.7∗	395.5∗	1696.3∗
O4–H		953.1	1653.2	1393.2	256.4	1649.6
C1–H		923.3	1623.3	1497.7	145.6	1643.3
C2–H		921.4	1621.5	1462.6	168.5	1631.1
C3–H		959.0	1659.1	1617.5	60.8	1678.3
C4–H		959.0	1659.1	1618.3	57.6	1675.9
C5–H		921.4	1621.5	1407.2	241.7	1648.9
C6–H		924.7	1624.8	1486.5	141.9	1628.4
**Irganox**	733.1					
O–H		911.7	1644.9	1406.9	238.0	1644.9
C1–H		1031.9	1765.0	1632.5	132.6	1765.0
C2–H		1028.5	1761.7	1622.7	140.7	1763.5
C3–H		1001.4	1734.5	1666.2	68.3	1734.5
C4–H		1000.4	1733.6	1701.7	31.8	1733.6
C5–H		942.8	1675.9	1576.0	99.9	1675.9
C6–H		963.1	1696.3	1525.5	170.8	1696.3
C7–H		991.1	1724.2	1668.2	52.4	1720.6

All in all, the O–H bond is more prone to deprotonation compared to the C–H sites and both EDTA and Irganox are more prone to express their antioxidant effect through the HAT than SET-PT or SPLET mechanisms.

∗ Rearrangement after deprotonation.

The IP of the Irganox model is higher compared to EDTA and it is just above 730 kJ/mol (Table 1 and Table S6). Furthermore, the PDE values cover a range between 911.7 and 1031.9 kJ/mol (calculated at the M06-2X/6–311++G(2d,2p) level of theory) where the lowest one belongs to the O–H bond and the strongest one is C1–H which is a benzylic hydrogen (Table 1). The most potent antioxidant site of the Irganox model is O–H followed by C5–H, C6–H, C7–H, C4–H, C3–H, C2–H, and C1–H. Thus, the strongest bonds are C1–H and C2–H which are benzylic hydrogens, while the weakest bonds are the O–H, C5–H, and C6–H. By comparing the ionization potentials of the compounds, it can be seen that EDTA has stronger electron donating ability than Irganox. Furthermore, the sum of the IP and PDE values further strengthen that EDTA has higher antioxidant potential compared to Irganox, and this also is in good agreement with the HAT results.

#### 3.2.3 Sequential proton loss electron transfer (SPLET)

The last antioxidant mechanism to consider is SPLET. It also includes two steps, a proton transfer followed by an electron transfer from the antioxidant to the radical. The values of proton affinity (PA) and electron transfer enthalpy (ETE) define the antioxidant potential for the studied compounds, the lower PA and ETE the better antioxidant. The computed PA and ETE values of the X–H (X = O or C) bonds of EDTA has been collected and compared (Table 1 and Table S7).

**Figure 5:**
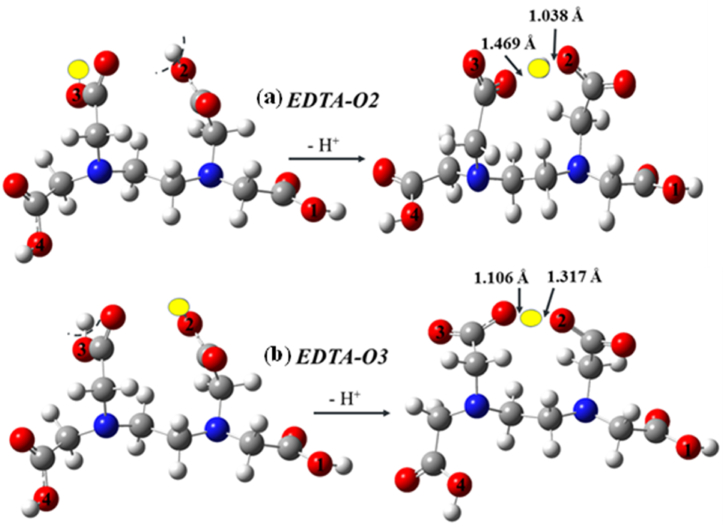
Optimized structures of ethylenediaminetetraacetic acid (EDTA) and the corresponding anionic species after proton loss in the first step of the SPLET mechanism in the cases of the (a) O2–H (EDTA-O2) and (b) O3–H (EDTA-O3) donor sites. The species have been computed at the M06-2X/6–311++G(2d,2p) level of theory in the gas phase and the corresponding specific geometric parameters are also shown (in Å).

In the case of EDTA, during the first step of SPL, in case of the deprotonation from O2–H and O3–H, the process leads to an interesting situation. Both deprotonated species rearrange in a way that the nearby proton holding carboxyl group will stabilize the system by forming a bridge with the group which just lost its proton (Figure 5 and Figure S4) and thus, the remaining proton will be shared between the two groups. Nevertheless, the corresponding PA and ETA values have also been computed and compared to C–H bonds, but the conclusions drawn by using these two cases have to consider the specificity of the mechanism. According to the calculated proton affinities for all, from highest to lowest the X–H sites within EDTA are ranked in the following order: C4–H, C3–H, C1–H, C6–H, C2–H, O1–H, C5–H, O4–H, O2–H, and O3–H. Thus, O–H sites are more prone to deprotonation, but in terms of ETE results, the C–H bonds have lower values compared to O–H bonds.

Due to the special situation (O2 and O3 in SPL), the order of the antioxidant potential (PA + ETE) in this mechanism is different compared to the HAT and SET-PT mechanisms. The antioxidant potential of C3–H and C4–H were the lowest among all bonds in case of HAT and SET-PT, but in this mechanism O2–H and O3–H are the least potent antioxidant sites.

In case of the Irganox model, PA values cover a range between 1406.9 (O–H) and 1701.7 (C4–H) kJ/mol calculated at the M06-2X/6–311++G(2d,2p) level of theory. As for ETE results of Irganox, the values start from 31.8 to 238.0 kJ/mol (Table 1). From the result, we can notice that the PA and ETE results indicate that the antioxidant potential of O–H is higher than C–H bonds which is in good agreement with the results of the HAT and SET-PT mechanisms.

### 3.3 Quantum Theory of Atoms in Molecules (QTAIM)

The electron density (ED) distribution obviously gives a valuable information on various molecular properties, with the chemical bonding at the first place [42, 43, 48]. The worth mentioning fact is that ED is the own property of the system in a given chemical and physical state or, in other words, it is a property being independent of any reference system or state in respect of the system/state under investigation. For that reason, the analysis of ED distribution, made according to QTAIM approach, seems to be an obvious choice when the bonding between individual atoms in a given molecule is analysed. For the purpose of our research, we analyse the X–H bonds in EDTA and Irganox model structure (Table S9, Figure S5, and Table S10, Figure S6).

Considering ρ values at BCPs it is important to point out that bonds can be compared only in subgroups of the same type in the context of atoms involved in their formation, which is due to the strong dependence of ρ on the type of atoms forming a given bond [45]. So, for EDTA, by comparing ρ in C–H bonds one can see that the values are relatively very close to each other, with the maximum difference of 0.01 a. u. That is in agreement with expectations, since all C–H bonds are chemically equivalent belonging to –CH_2_- groups. However, if referring to ρ at BCP as a measure of bonding strength, the lower value of that parameter corresponds to relatively weaker chemical bond. Therefore, the relatively weakest C–H bond in EDTA is that formed by C1 carbon atom (C1–H*). The C–H bonds with contribution of C4 and C5 are also close to C1 in this ranking. In opposite, relatively strongest ones are those with contribution of C6 and C2. Nevertheless, all C–H bonds are very similar to each other if ρBCP is considered, and it is rather difficult to indicate the one which clearly differs from others. A slightly different situation appears when analysing the delocalisation index. In this case the most effective electron sharing is observed for C4–H and C3–H in case of which C atoms structurally differs from other carbon atoms in EDTA due to their chemical neighbouring. Anyway, values of DI are in general close to each other due to chemical similarity of –CH_2_- groups. Analogous situation occurs in case of O–H bonds. Again, they all belong to carboxyl groups and have relatively similar electron density characteristics. Nevertheless, if comparing their QTAIM properties, the O2–H shall be considered relatively the weakest while O1–H seem to be the strongest (Table S9 and Figure S5).

Whist for Irganox system we have four types of C–H bonds if considering the chemical environment of parent carbon atoms. Thus, there is a terminal –CH_3_ group, –CH_2_- methylene group and the CH one belonging to the aromatic ring. In the case of the first subgroup the C7 seem to clearly differ from other terminal –CH_3_ groups due to the fact that it belongs to a methoxyl substituent. Electron density characteristics at proper BCPs indicate C7 as the one forming the strongest C–H bonds. Next are aromatic carbon atoms while the other C–H are relatively weaker and similar to each other. Ordering C–H bonds by delocalisation index, one may conclude that C7 and both aromatic C atoms form clearly more polarised C–H bonds, which is reflected by relatively lower values. In general, C–H bonds in tert-butyl groups are those which should dissociate as the first, when considering electron density distribution as the only indicator of bonding characterisation (Table S10 and Figure S6). There is only one O–H bond in Irganox model, so it cannot be compared with other this type bonds in that system. However, when comparing its QTAIM parameters with O–H bonds in EDTA, it seems to be stronger and less available for dissociation than O–H bonds in EDTA. This is not the case when BDEs are compared (338.5 kJ/mol *vs* 333.8 kJ/mol) and thus, the intrinsic characteristic of the O–H bonds slightly differ from those results. The reason behind is the stability of the formed radicals which prefers the hydrogen removal from Irganox over EDTA and that makes the process slightly more preferable for the former.

## 4 Conclusion

The target for this work is to study the antioxidant additives which can be used to protect polymers and prevent material deterioration caused by oxidative stress. In the future, the most active antioxidant additive will be selected, and its potential will also be explored in polymer synthesis. The antioxidant potential of two synthetic antioxidant additives including EDTA and Irganox model have been studied. The ability of these additives to donate hydrogen atoms to free radicals was compared by using computational tools. Based on DFT calculations employing two global hybrid functionals, the most likely radical scavenging sites were investigated. From the result, we noticed that an interesting situation happened including the hydrogen atom abstraction from a carboxyl group of EDTA initiated a successive C–C bond break, resulting in decarboxylation. Furthermore, the proton loss from O2–H and O3–H leads to both deprotonated species rearranges in a way that the nearby proton holding carboxyl group will stabilize the system by forming a bridge with the group which lost its proton and thus, the proton will be shared between the two groups. O–H bonds are more potent radical scavenging sites, but some C–H bonds also have significant antioxidant potential depending on their position in the structures. By comparing the lowest values of the computed BDE, IP, PDE, PA, and ETE of the studied structures, it can be concluded that EDTA has a higher antioxidant potential than Irganox. The most probable antioxidant mechanism of the studied species is the hydrogen atom transfer. Various additional molecular properties including the electron density (ED) distribution and others were studied by using Quantum Theory of Atoms in Molecules (QTAIM). While, when comparing its QTAIM parameters with O–H bonds in EDTA, it seems to be stronger and less available for dissociation than O–H bonds in EDTA, which is the case if only the intrinsic characteristic of the bonds are considered. But to compute BDE, the corresponding radical species have to be studied and thus, their stability also important. To fully understand the antioxidant activity, both, BDE and BCP are important to analyse and compare. The ability of these antioxidant additives has been compared by the experimental BDE values of regularly used polymers and it was found that bond dissociation enthalpy values of the polymers are higher than the lowest BDE values of EDTA and Irganox. Therefore, regardless of the order of the antioxidant potential of the different X–H bonds, the two studied species include such units which makes them excellent additives and thus, we are thinking about the future research directions will be about that, these additives can be used to protect pre-processed food products and polymeric packaging and prevent material deterioration caused by oxidative stress. Moreover, these additives also can be used as stabilizers during the polymerisation with the common polymers such as PVC.

## Author contribution statement

Dalal K. Thbayh, Marcin Palusiak, Béla Viskolcz, Béla Fiser: Conceived and designed the experiments; Performed the experiments; Analyzed and interpreted the data; Contributed reagents, materials, analysis tools or data; Wrote the paper.

## Data availability statement

Data included in article/supplementary material/referenced in article.

## Declaration of competing interest

The authors declare that they have no known competing financial interests or personal relationships that could have appeared to influence the work reported in this paper.
