# Supplementary material for: Comparative study of the antioxidant capability of EDTA and Irganox
Source: Heliyon. 2023 May 13;9(5):e16064. doi: 10.1016/j.heliyon.2023.e16064 (PMC10205517; doi:10.1016/j.heliyon.2023.e16064)
Supplement: Multimedia component 1 [file mmc1.docx]

**Comparative Study of the Antioxidant Capability of EDTA and Irganox**

**Dalal K. Thbayh^1,2,3^, Marcin Palusiak^4^, Béla Viskolcz^1,3^, Béla Fiser^1,3,4,5,*^**

^1^ Institute of Chemistry, University of Miskolc, 3515 Miskolc-Egyetemváros, Hungary

^2^ Polymer Research Center, University of Basrah, Basrah, Iraq

^3^ Higher Education and Industrial Cooperation Centre, University of Miskolc, 3515 Miskolc-Egyetemváros, Hungary

^4^ Department of Physical Chemistry, Faculty of Chemistry, University of Lodz, 90-236 Lodz, Poland

^5^ Ferenc Rakoczi II Transcarpathian Hungarian College of Higher Education, 90200 Beregszász, Transcarpathia, Ukraine

*kemfiser@uni-miskolc.hu

**Supplementary Information**

**List of abbreviations**

**HAT:** hydrogen atom transfer

**BDE:**  bond dissociation enthalpy

**SET-PT**: single electron transfer-proton transfer

**PA:** proton affinity

**ETE:** electron transfer enthalpy

**SPLET**: sequential proton loss electron transfer

**IP:** ionization potential

**PDE:** proton dissociation enthalpy

**EDTA**: ethylenediaminetetraacetic acid


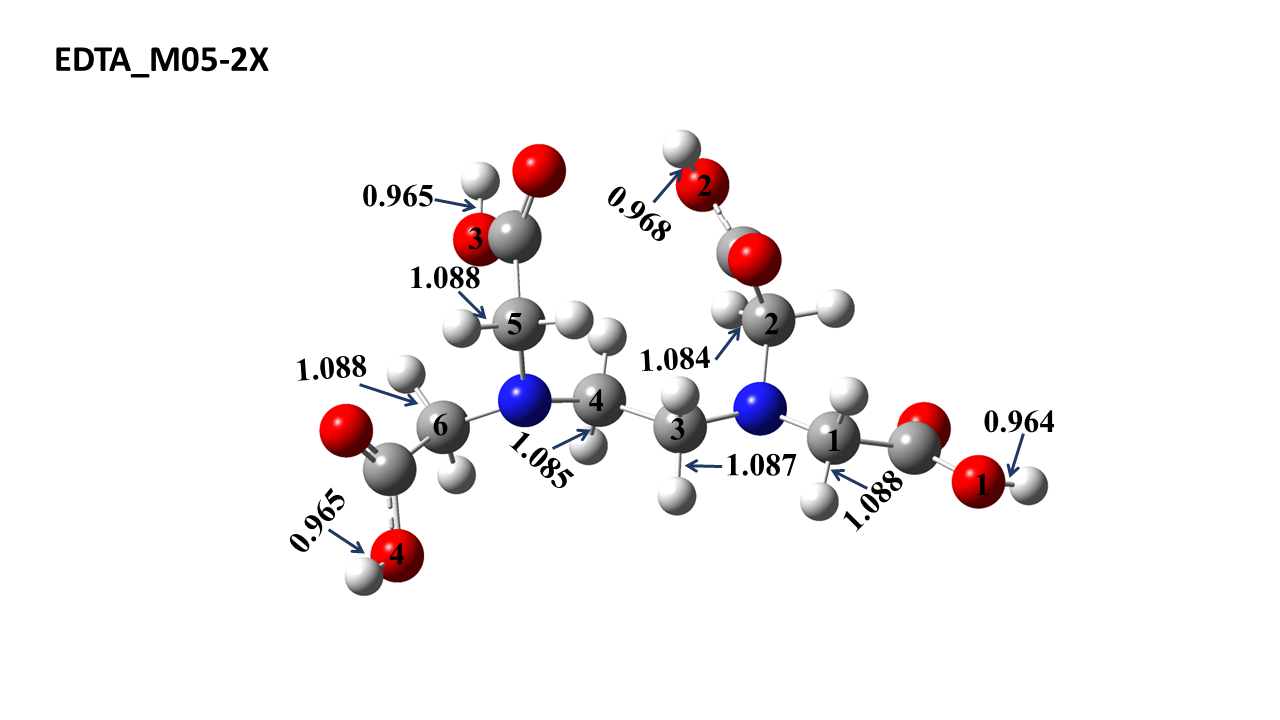


***Figure S1*** Optimized geometry of the studied synthetic antioxidant additive ethylenediaminetetraacetic acid (EDTA). Geometry optimization has been carried out at the M05-2X/6-311++G(2d,2p) level of theory in gas phase, and the corresponding bond lengths (in Å) are also shown.


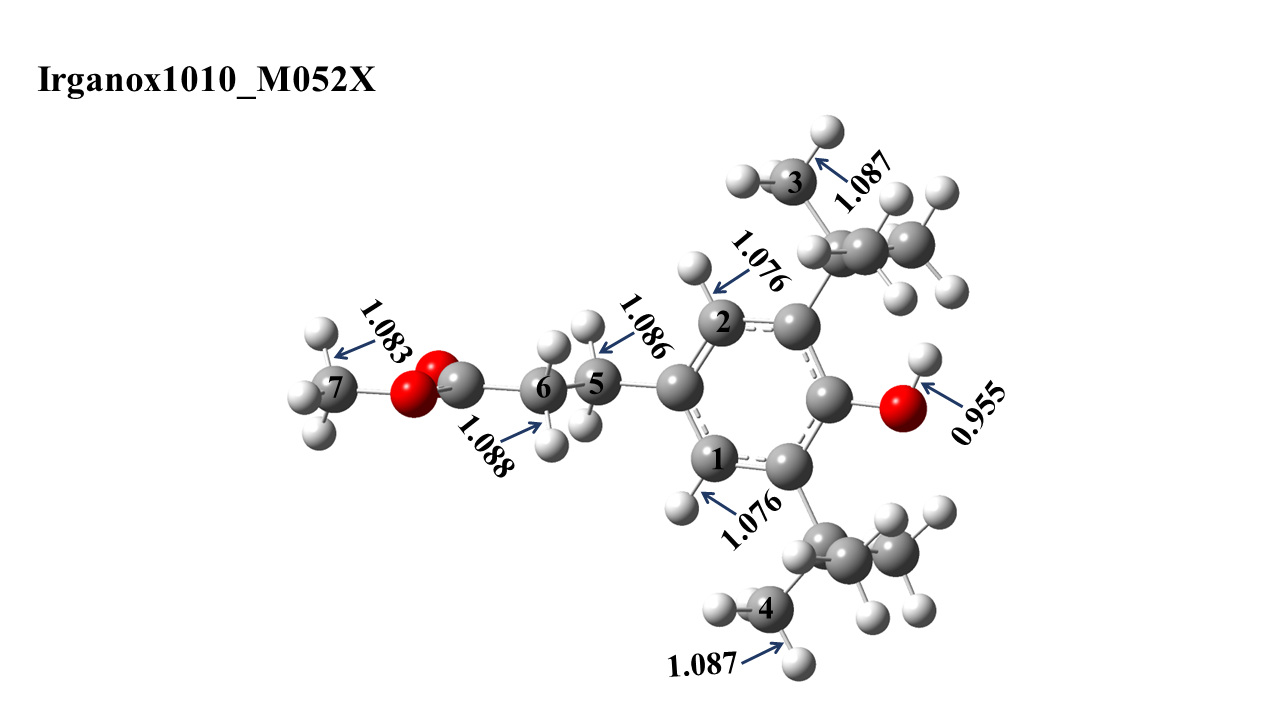


**Figure S2** Optimized geometry of the studied Irganox model. Geometry optimization has been carried out at the M05-2X/6-311++G(2d,2p) level of theory in gas phase, and the corresponding bond lengths (in Å) are also shown.

***Table S1*** Bond dissociation enthalpy (BDE) (in kJ/mol) for all unique C-H and O-H bonds in ethylenediaminetetraacetic acid (EDTA) at the M05-2X/6-311++G(2d,2p) level of theory in gas phase.

| EDTA | M05-2X/  6-311++G(2d,2p) |
| --- | --- |
| X-H  positions | BDE (kJ/mol) |
| O1-H | 362.0 |
| O2-H | 361.1 |
| O3-H | 345.4 |
| O4-H | 342.9 |
| C1-H | 328.0 |
| C2-H | 321.4 |
| C3-H | 368.4 |
| C4-H | 365.7 |
| C5-H | 317.5 |
| C6-H | 313.1 |

***Table S2*** Bond dissociation enthalpy (BDE) (in kJ/mol) for all unique C-H and O-H bonds in ethylenediaminetetraacetic acid (EDTA) at the M06-2X/6-311++G(2d,2p) level of theory in gas phase.

| EDTA | M06-2X/  6-311++G(2d,2p) |
| --- | --- |
| X-H | BDE  (kJ/mol) |
| O1-H | 354.0 |
| O2-H | 351.8 |
| O3-H | 340.4 |
| O4-H | 338.5 |
| C1-H | 330.3 |
| C2-H | 323.6 |
| C3-H | 367.2 |
| C4-H | 364.8 |
| C5-H | 316.7 |
| C6-H | 317.3 |

***Table S3*** Bond dissociation enthalpy (BDE) (in kJ/mol) for all unique C-H and O-H bonds in the studied Irganox model at the M05-2X/6-311++G(2d,2p) level of theory in gas phase.

| Irganox | M05-2X/  6-311++G(2d,2p) |
| --- | --- |
| X-H  positions | BDE  (kJ/mol) |
| O-H | 331.7 |
| C1-H | 458.5 |
| C2-H | 459.9 |
| C3-H | 423.0 |
| C4-H | 422.2 |
| C5-H | 358.3 |
| C6-H | 383.3 |
| C7-H | 412.3 |

***Table S4*** Bond dissociation enthalpy (BDE) (in kJ/mol) for all unique C-H and O-H bonds in the studied Irganox model at the M06-2X/6-311++G(2d,2p) level of theory in gas phase.

| Irganox | M06-2X/  6-311++G(2d,2p) |
| --- | --- |
| X-H  positions | BDE  (kJ/mol) |
| O-H | 333.8 |
| C1-H | 454.0 |
| C2-H | 450.6 |
| C3-H | 423.4 |
| C4-H | 422.5 |
| C5-H | 364.8 |
| C6-H | 385.2 |
| C7-H | 413.1 |


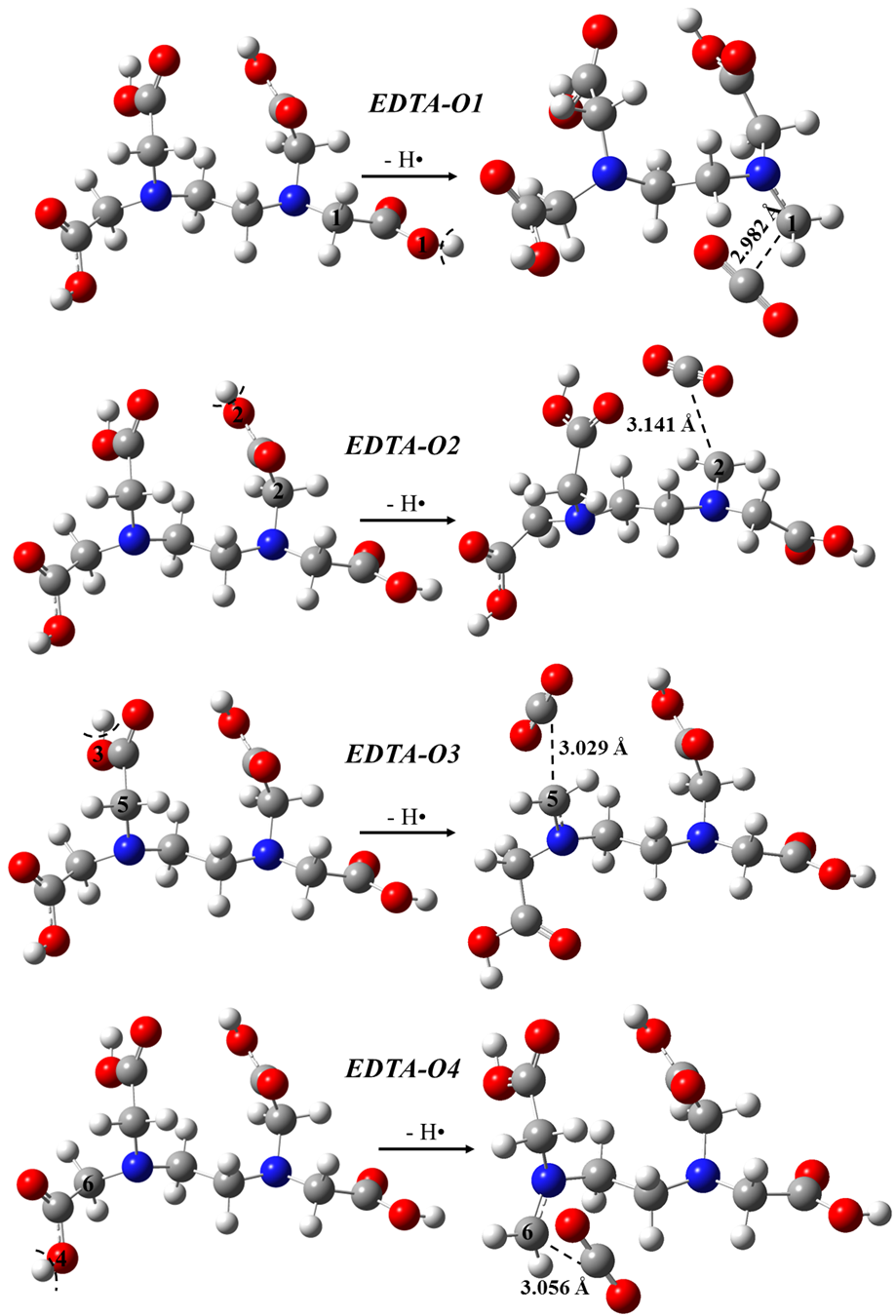


**Figure S3** Optimized structures of ethylenediaminetetraacetic acid (EDTA) and the corresponding radical species after H-atom transfer in the hydrogen atom transfer (HAT) mechanism in case of the O1-H, O2-H, O3-H, and O4-H sites. The species have been computed at the M05-2X/6-311++G(2d,2p) level of theory in the gas phase and specific geometrical parameters are also shown (in Å).

**Table S5** The ionization potential (IP) and proton dissociation enthalpy (PDE) values in kJ/mol for ethylenediaminetetraacetic acid (EDTA) calculated at the M05-2X/6-311++G(2d,2p) level of theory in gas phase.

| Compound | IP | PDE | IP+PDE |
| --- | --- | --- | --- |
| EDTA | 702.1 |  |  |
| O1-H |  | 967.7 | 1669.7 |
| O2-H |  | 959.7 | 1661.8 |
| O3-H |  | 959.9 | 1662.0 |
| O4-H |  | 962.7 | 1664.7 |
| C1-H |  | 918.8 | 1620.9 |
| C2-H |  | 922.4 | 1624.5 |
| C3-H |  | 975.0 | 1677.1 |
| C4-H |  | 960.3 | 1662.3 |
| C5-H |  | 922.5 | 1624.5 |
| C6-H |  | 922.0 | 1624.1 |

**Table S6** The ionization potential (IP) and proton dissociation enthalpy (PDE) values in kJ/mol for the studied Irganox model calculated at the M05-2X/6-311++G(2d,2p) level of theory in gas phase.

| Compound | IP | PDE | IP+PDE |
| --- | --- | --- | --- |
| Irganox | 730.5 |  |  |
| O-H |  | 915.2 | 1645.7 |
| C1-H |  | 1041.6 | 1772.1 |
| C2-H |  | 1043.4 | 1773.9 |
| C3-H |  | 1006.5 | 1737.0 |
| C4-H |  | 1005.6 | 1736.2 |
| C5-H |  | 941.8 | 1672.3 |
| C6-H |  | 966.8 | 1697.3 |
| C7-H |  | 995.7 | 1726.3 |

**Table S7** The proton affinities (PAs) and electron transfer enthalpies (ETE) in kJ/mol for ethylenediaminetetraacetic acid (EDTA) calculated at the M05-2X/6-311++G(2d,2p) level of theory in gas phase.

| EDTA | PA | ETE | PA+ETE |
| --- | --- | --- | --- |
| O1-H | 1407.1 | 268.8 | 1676.0 |
| O2-H | 1321.8 | 385.7 | 1707.4 |
| O3-H | 1295.5 | 401.5 | 1697.0 |
| O4-H | 1388.7 | 271.1 | 1659.8 |
| C1-H | 1504.3 | 137.8 | 1642.0 |
| C2-H | 1466.4 | 165.7 | 1632.1 |
| C3-H | 1624.4 | 58.0 | 1682.4 |
| C4-H | 1624.2 | 55.5 | 1679.7 |
| C5-H | 1402.6 | 248.2 | 1650.8 |
| C6-H | 1483.6 | 143.4 | 1627.0 |

**Table S8** The proton affinities (PAs) and electron transfer enthalpies (ETE) in kJ/mol for the studied Irganox model calculated at the M05-2X/6-311++G(2d,2p) level of theory in gas phase.

| Irganox | PA | ETE | PA+ETE |
| --- | --- | --- | --- |
| O-H | 1409.5 | 236.2 | 1645.7 |
| C1-H | 1639.6 | 132.5 | 1772.1 |
| C2-H | 1626.5 | 147.4 | 1773.9 |
| C3-H | 1669.6 | 67.4 | 1737.0 |
| C4-H | 1707.7 | 28.5 | 1736.2 |
| C5-H | 1578.5 | 93.3 | 1671.9 |
| C6-H | 1526.3 | 171.0 | 1697.3 |
| C7-H | 1674.7 | 51.6 | 1726.3 |


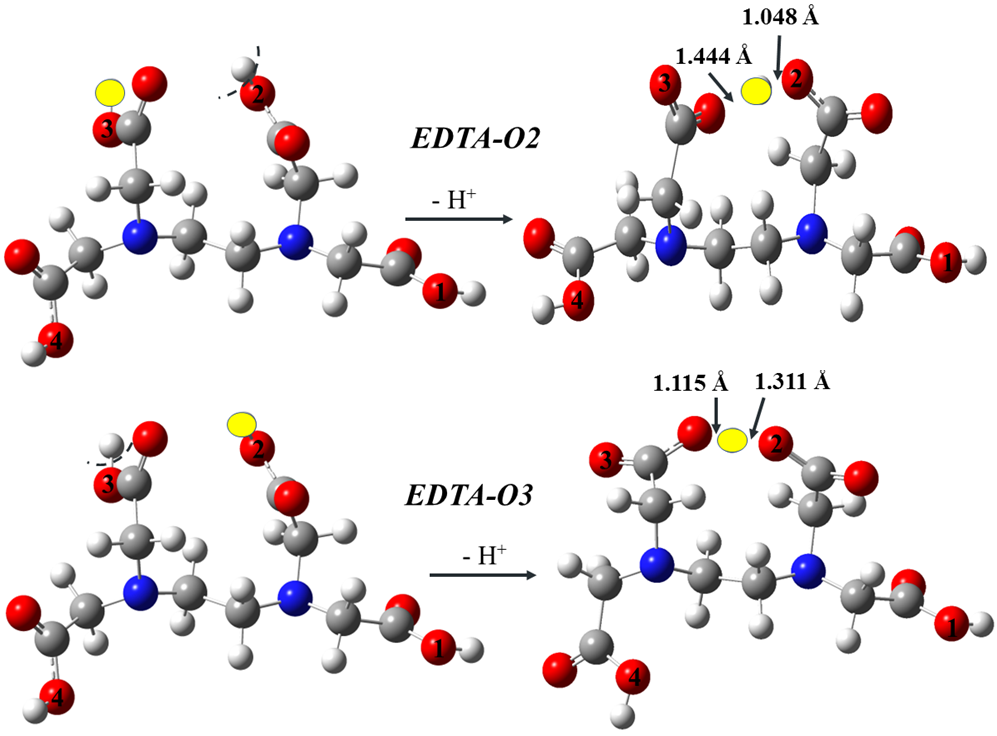


***Figure S4*** Optimized structures of ethylenediaminetetraacetic acid (EDTA) and the corresponding anionic species after proton loss in the first step of the SPLET mechanism in the cases of the O2-H and O3-H donor sites. The species have been computed at the M05-2X/6-311++G(2d,2p) level of theory in the gas phase and the corresponding specific geometric parameters are also shown (in Å).

**Table S9** contains numerical data from QTAIM analysis for ethylenediaminetetraacetic acid (EDTA) including electron density rho and its laplacian, both estimated in C-H and O-H bond critical points and, additionally, delocalisation index values obtained for atoms forming corresponding bonds. All of them calculated at the M06-2X/6-311++G(2d,2p) level of theory.

| **Atoms** | **ρ** | $\boldsymbol{\nabla}$**^2^ρ** | **DI** |
| --- | --- | --- | --- |
| C1-H* | 0.280001 | -1.002466 | 8.8968306656E-01 |
| C4-H* | 0.283597 | -1.025099 | 8.8324665323E-01 |
| C1-H | 0.284036 | -1.034942 | 9.0127254185E-01 |
| C5-H | 0.284681 | -1.045114 | 8.8381409902E-01 |
| C3-H* | 0.285094 | -1.034535 | 8.8577800776E-01 |
| C2-H* | 0.285843 | -1.052664 | 8.8777652519E-01 |
| C6-H | 0.286481 | -1.051141 | 8.9921937501E-01 |
| C3-H | 0.286600 | -1.051694 | 9.1546359245E-01 |
| C4-H | 0.287301 | -1.058186 | 9.1484677679E-01 |
| C5-H* | 0.287964 | -1.064637 | 8.9251998794E-01 |
| C2-H | 0.288877 | -1.068092 | 9.0523648141E-01 |
| C6-H* | 0.289695 | -1.076814 | 9.1110536099E-01 |
| O2-H | 0.358137 | -2.711776 | 5.6098468641E-01 |
| O3-H | 0.363333 | -2.716107 | 6.0012999537E-01 |
| O4-H | 0.364567 | -2.718208 | 6.0915438007E-01 |
| O1-H | 0.365002 | -2.724619 | 6.0788724635E-01 |

H represents hydrogen atoms which were removed from the structure to study the antioxidant potential, while H* represents the second hydrogen atom bonding to the C atom.


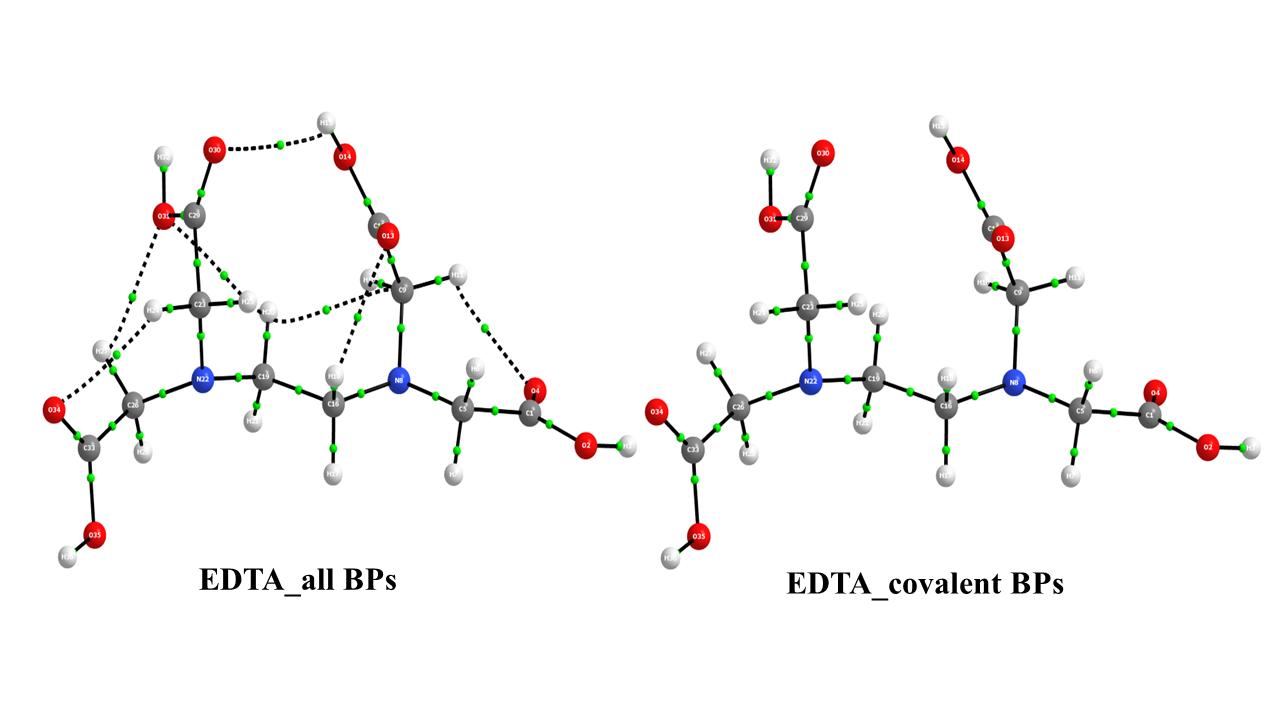


***Figure S5*** Optimized structures of ethylenediaminetetraacetic acid (EDTA) have been computed at the M06-2X/6-311++G(2d.2p) level of theory in the gas phase and bond critical points are determined.

**Table S10** contains numerical data from QTAIM analysis for the studied Irganox model including electron density rho and its laplacian. both estimated in C-H and O-H bond critical points and. additionally. delocalisation index values obtained for atoms forming corresponding bonds. All of them calculated at the M06-2X/6-311++G(2d.2p) level of theory.

| **Atoms** | **ρ** | $\boldsymbol{\nabla}$**^2^ρ** | **DI** |
| --- | --- | --- | --- |
| C4*-H** | 0.278352 | -0.992412 | 9.5225794310E-01 |
| C4**-H*** | 0.278382 | -0.992601 | 9.5234805879E-01 |
| C4**-H** | 0.278958 | -0.997137 | 9.5585095941E-01 |
| C4*-H*** | 0.279124 | -0.998187 | 9.5546246549E-01 |
| C4-H** | 0.279557 | -1.000444 | 9.4608456294E-01 |
| C3*-H* | 0.279643 | -1.000953 | 9.4938679047E-01 |
| C3**-H* | 0.279656 | -1.001029 | 9.4929327321E-01 |
| C4-H* | 0.279723 | -1.001457 | 9.4561788269E-01 |
| C3**-H*** | 0.279725 | -1.003802 | 9.4191506545E-01 |
| C6-H* | 0.279752 | -1.000897 | 9.2698367886E-01 |
| C6-H | 0.279798 | -1.001219 | 9.2604069398E-01 |
| C3*-H** | 0.279935 | -1.005352 | 9.4200291297E-01 |
| C4-H | 0.280188 | -1.004712 | 9.4703962775E-01 |
| C3-H | 0.280245 | -1.004791 | 9.4624073938E-01 |
| C3-H** | 0.280618 | -1.007803 | 9.4378334563E-01 |
| C3**-H** | 0.280715 | -1.008719 | 9.4910992866E-01 |
| C3-H* | 0.280787 | -1.008882 | 9.4344346434E-01 |
| C3*-H*** | 0.280840 | -1.009543 | 9.4896644378E-01 |
| C5-H | 0.282924 | -1.022679 | 9.3087078015E-01 |
| C5-H* | 0.282937 | -1.022592 | 9.3005594741E-01 |
| C4*-H* | 0.285049 | -1.041432 | 9.3654312489E-01 |
| C4**-H* | 0.285052 | -1.041178 | 9.3615319974E-01 |
| C1-H | 0.289418 | -1.082726 | 9.3075871806E-01 |
| C2-H | 0.289425 | -1.082379 | 9.3161235110E-01 |
| C7-H | 0.289985 | -1.082941 | 9.2573790284E-01 |
| C7-H* | 0.290039 | -1.083393 | 9.2541809000E-01 |
| C7-H** | 0.292225 | -1.102344 | 9.3775981695E-01 |
| O-H | 0.376393 | -2.809381 | 5.8326339741E-01 |

H represents hydrogen atoms which were removed from the structure to study the antioxidant potential, while H* and H** represents the second and third hydrogen atoms bonding to the same C atom. C* and C** are the second and third C atoms in the tert-butyl group of the structure, while H*, H**, and H*** are the atoms belonging to them.

 
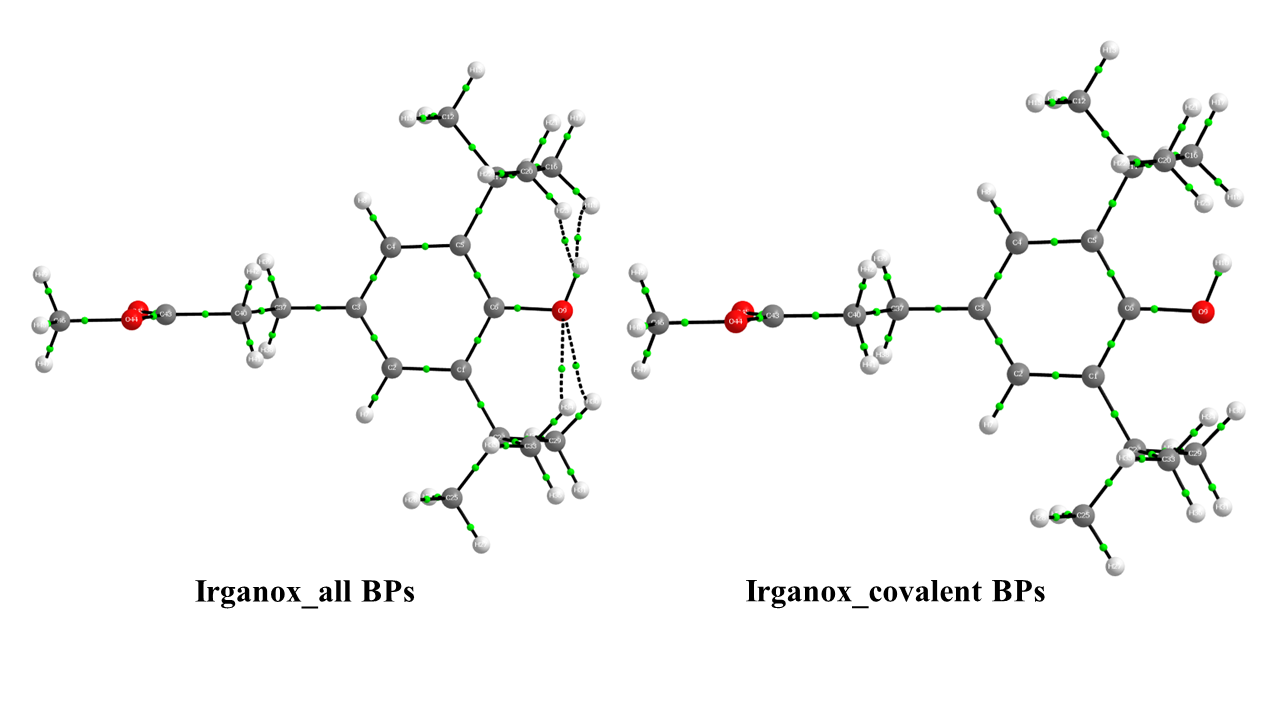


***Figure S6*** Optimized geometry of the studied Irganox model. Geometry optimization has been carried out at the M06-2X/6-311++G(2d.2p) level of theory in gas phase and bond critical points are determined.
